# Keratoacanthoma in the External Auditory Canal

**DOI:** 10.7759/cureus.16873

**Published:** 2021-08-04

**Authors:** Javier Ash, Raju Limbu, Victoria Alexander, David Selvadurai

**Affiliations:** 1 General Surgery, Princess Alexandra Hospital, Harlow, GBR; 2 Otolaryngology, St. George's University Hospital, London, GBR

**Keywords:** keratoacanthoma, otology, external auditory canal, severe otalgia, otorrhoea

## Abstract

A 63-year-old male presented with a prolonged history of bilateral ear discharge, otalgia, and hearing loss. The patient required a significant number of investigations prior to obtaining a diagnosis. Subsequent investigations identified keratoacanthoma affecting the external ear canal (EAC). There has been no case report of keratoacanthoma within the EAC till now. The only risk factor identified for the development of keratoacanthoma, in this case, was the frequent use of earbuds and subsequent long-term trauma associated with a retained bud. The histology of keratoacanthoma is difficult to differentiate from that of squamous cell carcinoma but this is essential for the ear, nose, throat (ENT) multi-disciplinary team.

## Introduction

A keratoacanthoma is a rapidly growing benign epithelial tumour that originates from pilosebaceous glands (hair follicles) [1]. It usually presents as a firm, cone-shaped nodule with a keratin-filled crater and typically affects sun-exposed regions of light-skinned middle-aged and elderly individuals [1]. Classically it will grow to a size of 1-2 cm and then spontaneously involute.

Keratoacanthomata have three stages of growth: a proliferative stage that is smooth and enlarging and lasts two to four weeks, a mature phase where it is the classical dome-shaped nodule with a keratinous core, and a third phase which is characterized by the regression and explosion of the keratinous plug resulting in a hypopigmented scar [2]. A keratoacanthoma is considered to be a low-grade variant of squamous cell carcinoma. Due to its rapid growth and histological appearance, differentiating is difficult and surgical excision is usually performed before involution occurs [2]. Histopathological differentiation is challenging but can be performed using various markers, which allow differentiation between the two entities [3].

Causative factors for Keratoacanthomata have not been clearly identified. Its pre-disposition for sun-exposed areas and in patients with xeroderma pigmentosum lend support to theories aligning the degree of UV exposure to its development [2]. Alternatively, the increased incidence on or near skin grafts also suggests trauma as a probable cause [4].

A keratoacanthoma within otorhinolaryngology is rare [5]. Similar masses have, at times, been diagnosed as cutaneous neuroendocrine carcinomas or other tumour types such as melanomas, which may require extensive treatment. Hence, a pathological examination is important for diagnosis [6]. There have been no case reports describing keratoacanthomata within the external auditory canal (EAC).

## Case presentation

A 63-year-old male presented to the emergency ear, nose and throat (ENT) clinic complaining of bilateral ear discharge, more pronounced in the right ear, associated otalgia, and hearing loss for several months. The patient had no other medical problems. 

The patient was treated in primary care with a seven-day course of amoxicillin. Pain and hearing loss persisted and thus the patient was referred to the otology department. 

On initial examination, a cotton wool bud was found in the right EAC and removed via forceps. Following microsuction of the right and left EACs, evidence of bilateral otitis externa with a right ear canal polyp was seen. The patient was prescribed a 10-day course of betnesol and ciprofloxacin drops.

**Figure 1:**
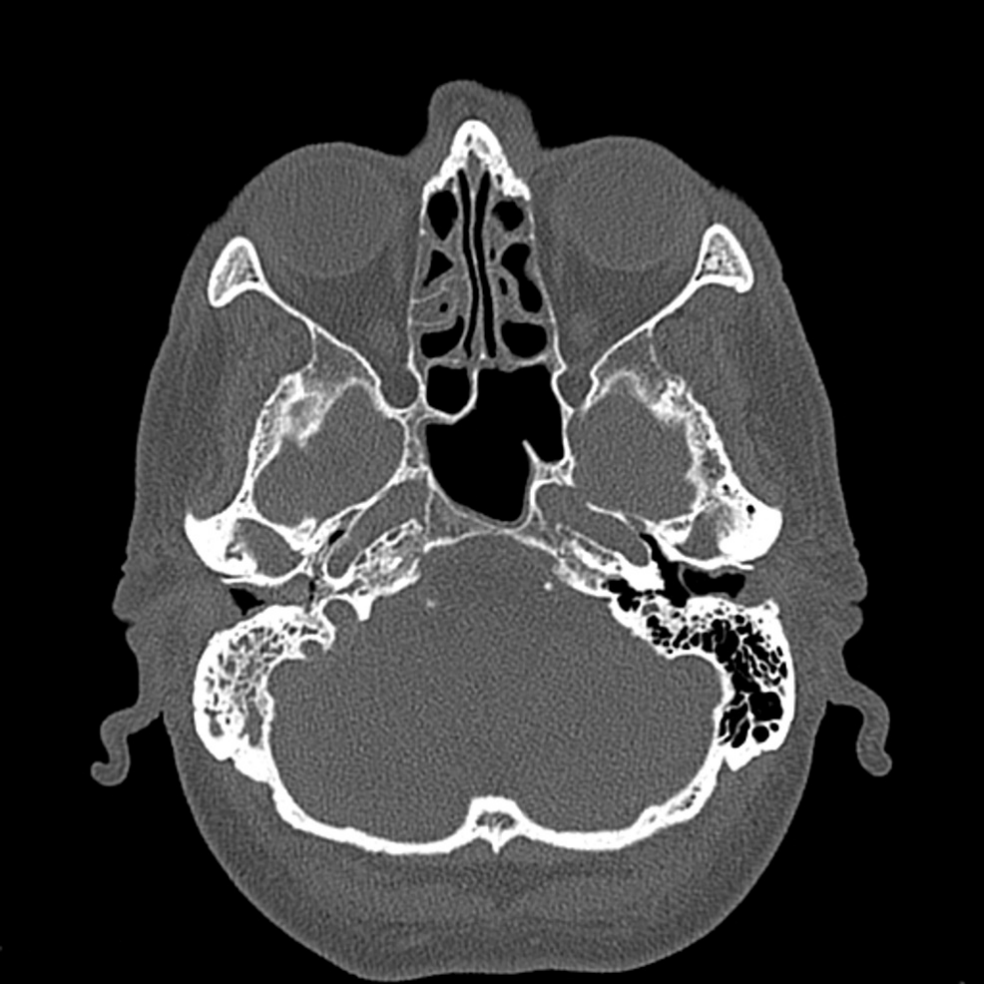
Axial Computer Tomography of the Internal Auditory Meatus

On review two weeks later, the patient continued to complain of a mild degree of hearing loss and associated non-pulsatile tinnitus. On examination, the right EAC was observed to be narrowed and a polyp was still present. The left otitis externa had resolved. An audiogram showed a mixed conductive and sensorineural hearing loss on the right side and mild high-frequency hearing loss on the left. Due to the chronicity of the patient's symptoms, a CT scan of the temporal bones was completed. This demonstrated: chronic bilateral thickening of the EACs, right middle ear inflammatory disease, and subtle ossicular erosions (Figure 1). The patient was listed for examination plus biopsy under anaesthesia to better delineate the cause of his ongoing symptoms.

Under anaesthesia, thick inflammatory tissue was noted arising from the inferior suture line and anterior external auditory canal wall. The pars tensa was just visible posteriorly. Multiple biopsies were taken and sent for histology and microbiology. The right ear was packed with two pieces of bismuth iodoform paraffin paste (BIPP) packs. 

On review in the clinic, the packing was removed, revealing that the right EAC was still filled with polypoid material and thick pus. The microbiology results confirmed *Pseudomonas aeruginosa* and *Serratia marcescens*. Histology showed a hyperkeratotic and squamoproliferative lesion showing moderate-severe dysplasia. The ear was re-packed with Tri-Adcortyl soaked ribbon gauze (active ingredients: triamcinolone acetonide, neomycin sulphate, gramicidin, and nystatin) and the patient was re-prescribed ciprofloxacin and started on flucloxacillin as per sensitivities and listed for deep biopsies of the right ear under general anaesthesia.

On examination, no significant bony erosion was seen. A very thick and inflamed EAC epithelium and a thickened tympanic membrane were observed again. The tympanomeatal epithelium was fully excised down to the bone of the EAC and circumferentially to the edge of the annulus. Histological analysis showed skin hyperkeratosis and parakeratosis with epithelial budding. Focally, deep keratin pearls were identified. The dermis was fibrotic and minimally inflamed. These findings were consistent with regressed/regressing keratoacanthoma.

Over the next three weeks, the patient's ear discharge settled without further treatment. The patient continued to be under regular review in the outpatient ENT clinic and the initial presenting symptoms did not return over the period of a year. and Subsequently, he was discharged back to primary care.

## Discussion

Keratoacanthomata have been rarely reported in ENT literature and this case is the first reported case affecting the EAC. The patient presented with symptoms common within ENT clinics and had no significant history that would elucidate that a keratoacanthoma could be the cause of his symptoms. Differentials for the presentation included cholesteatoma, necrotising otitis externa and tumours such as basal cell carcinoma, melanoma and lymphoma [6]. Early clinical differentiation of these conditions is difficult but important. One risk factor in this case for the development of keratoacanthoma was the frequent use of ear buds and the unknown length of time the discovered bud had been present, which may have led to continual micro-traumas in the EAC. Though keratoacanthoma is usually removed in its entirety in easily exposed regions, its discovery in the EAC leads to some difficulties due to its close relation to squamous cell carcinoma. Due to the improvement in the patient's symptoms and imaging not suggestive of a malignant process, close observation of the lesion was opted for instead of subjecting the patient to a likely large and possibly unnecessary surgical procedure.

## Conclusions

We describe a case of keratoacanthoma within the EAC. The only recorded risk factor for this was the frequent use of earbuds and, therefore, its development is more likely to be associated with trauma rather than UV exposure. Although already discouraged by ENT surgeons, this case provides further evidence to discourage the use of earbuds in the general public. Furthermore, ENT clinicians should consider keratoacanthomata in a chronically discharging ear with a background history of trauma or the discovery of a long-term EAC foreign body, as these cases may be difficult to distinguish from squamous cell carcinoma or necrotising otitis externa. Both clinicians and pathologists should include keratoacanthomata in the list of differential diagnoses in chronically discharging ears.
